# Supplementary material for: Course of recovery of respiratory muscle strength and its associations with exercise capacity and handgrip strength: A prospective cohort study among survivors of critical illness
Source: PLoS One. 2023 Apr 13;18(4):e0284097. doi: 10.1371/journal.pone.0284097 (PMC10101425; doi:10.1371/journal.pone.0284097)
Supplement: S1 Table — SD: Standard Deviation, LOS: Length Of Stay, IQR: Interquartile Range, MV: Mechanical Ventilation, SNAQ65+: Short Nutritional Assessment Questionnaire 65+, PImax: maximum static inspiratory mouth pressure, PEmax: maximum static expiratory mouth pressure, GS: Grip Strength, TMST: Two-Minute Step Test. * independent samples t-test ‡ Mann Whitney U test. (PDF) [file pone.0284097.s001.pdf]

S1 Table: Sensitivity analysis: complete follow-up versus incomplete follow-up

Variable	Complete visits (n=30)	Incomplete visits (n=29)	Comparison
**Age (median/IQR)**	58 (50-64)	64 (58-69)	p 0.03*
**Gender (n, %)**<br>• **Male**<br>• **Female**	 22 (73.3)<br>8 (26.7)	 16 (55.2)<br>13 (44.8)	
**ICU LOS (median/IQR)**	15 (5-21)	10 (8-21)	p 0.75‡
**Hospital LOS (median/IQR)**	41 (23-56)	30 (20-49)	p 0.20‡
**MV days (median/IQR)**	12 (4-18)	10 (6-14)	p 0.85‡
**Admission category (n, %)**<br>• **Acute**<br>• **Elective**	 21 (70.0)<br>9 (30.0)	 20 (69.0)<br>9 (31.0)	
**Discharge location**<br>• **Home**<br>• **Rehabilitation facility**	 23 (76.7)<br>7 (23.3)	 20 (69.0)<br>9 (31.0)	
**SNAQ65+**<br>• **Green**<br>• **Orange**<br>• **Red**	 3 (10.0)<br>4 (13.3)<br>23 (76.7)	 1 (3.4)<br>2 (6.9)<br>26 (89.7)	
**Baseline outcome data**<br><br>**PImax (mean % predicted, SD)**<br>**PEmax (median % predicted, IQR)**<br>**GS (mean % predicted, SD)**<br>**TMST (median steps, IQR)**	 <br>67.9 (23.3)<br>69.6 (26.4)<br>71.8 (26.0)<br>57 (31)	 <br>65.5 (21.0)<br>81.2 (42.8)<br>72.3 (30.0)<br>53 (30)	 <br>p 0.74*<br>p 0.58‡<br>p 0.85*<br>p 0.46‡

SD: Standard Deviation, LOS: Length Of Stay, IQR: Interquartile Range, MV: Mechanical Ventilation, SNAQ65+: Short Nutritional Assessment Questionnaire 65+, PImax: maximum static inspiratory mouth pressure, PEmax: maximum static expiratory mouth pressure, GS: Grip Strength, TMST: Two-Minute Step Test

* independent samples t-test ‡ Mann Whitney U test
