# Supplementary material for: Course of recovery of respiratory muscle strength and its associations with exercise capacity and handgrip strength: A prospective cohort study among survivors of critical illness
Source: PLoS One. 2023 Apr 13;18(4):e0284097. doi: 10.1371/journal.pone.0284097 (PMC10101425; doi:10.1371/journal.pone.0284097)
Supplement: S2 Table — SD: Standard Deviation, LOS: Length Of Stay, IQR: Interquartile Range, MV: Mechanical Ventilation, SNAQ65+: Short Nutritional Assessment Questionnaire 65+, PImax: maximum static inspiratory mouth pressure, PEmax: maximum static expiratory mouth pressure, GS: Grip Strength, TMST: Two-Minute Step Test. * Significant between group difference, as tested with the independent samples Mann Whitney U test. (PDF) [file pone.0284097.s002.pdf]

S2 Table. Sensitivity analysis: REACH versus usual care

Variable	REACH (n=19)	Usual care (n=40)	Comparison
**Age (median/IQR)**	63 (56-65)	60 (52-67)	p 0.603
**Gender (n, %)**			
• **Male**	14 (73.7)	24 (60.0)	
• **Female**	5 (26.3)	16 (40.0)	
**ICU LOS (median/IQR)**	10 (5-21)	14 (9-22)	p 0.155
**Hospital LOS (median/IQR)**	23 (14-35)	42 (25-66)	p 0.003*
**MV days (median/IQR)**	8 (4-14)	13 (5-19)	p 0.245
**Admission category (n, %)**			
• **Acute**	17 (89.5)	24 (60.0)	
• **Elective**	2 (10.5)	16 (40.0)	
**Discharge location**			
• **Home**	19 (100)	24 (60.0)	
• **Rehabilitation facility**	-	16 (40.0)	
**SNAQ65+**			
• **Green**	1 (5.3)	3 (7.5)	
• **Orange**	2 (10.5)	4 (10.0)	
• **Red**	16 (84.2)	33 (82.5)	

SD: Standard Deviation, LOS: Length Of Stay, IQR: Interquartile Range, MV: Mechanical Ventilation, SNAQ65+: Short Nutritional Assessment Questionnaire 65+, PImax: maximum static inspiratory mouth pressure, PEmax: maximum static expiratory mouth pressure, GS: Grip Strength, TMST: Two-Minute Step Test

* Significant between group difference, as tested with the independent samples Mann Whitney U test
