# Supplementary material for: Course of recovery of respiratory muscle strength and its associations with exercise capacity and handgrip strength: A prospective cohort study among survivors of critical illness
Source: PLoS One. 2023 Apr 13;18(4):e0284097. doi: 10.1371/journal.pone.0284097 (PMC10101425; doi:10.1371/journal.pone.0284097)
Supplement: S3 Table — Results of linear mixed model analysis (LMM) p > 0.05 between REACH versus usual care at any timepoint for all variables. β: beta regression coefficient, CI: Confidence Interval, MIP: maximum static inspiratory pressure, MEP: maximum static expiratory pressure, FEC: Functional exercise capacity, expressed in total steps per 2 minutes (two-minute step test), HGS: handgrip Strength, TMST: Two-Minute Step Test, Kg: kilogram. (PDF) [file pone.0284097.s003.pdf]

S3 Table. Sensitivity analysis: difference in regression coefficients REACH versus usual care

Variable	Usual care (n=40)			REACH (n=19)		
**MIP % predicted cmH2O β (95% CI)**	65.6 (56.1 to 75.0)	93.0 (83.3 to 103.0)	97.0 (87.4 to 107.0)	4.9 (-12.1 to 21.9)	-0.8 (-16.8 to 15.2)	3.2 (-13.9 to 20.2)
**MEP % predicted cmH2O β (95% CI)**	78.9 (69.1 to 88.7)	100.9 (91.2 to 111.0)	104.3 (94.5 go 114.1)	-1.4 (-18.8 to 16.0)	-2.8 (-19.2 to 13.5)	-1.2 (-18.6 to 16.2)
**FEC β (95% CI)**	59.4 (48.6 to 70.2)	81.6 (71.6 to 91.7)	91.3 (80.5 to 102.1)	-5.5 (-23.3 to 12.4)	0.74 (-16.3 to 17.8)	-4.7 (-22.9 to 13.6)
**HGS % predicted kg β (95% CI)**	73.1 (64.2 to 82.0)	94.1 (84.9 to 103.2)	102.7 (93.5 to 111.9)	-3.6 (-19.0 to 11.8)	3.2 (-12.0 to 18.4)	1.9 (-14.3 to 18.2)

Results of linear mixed model analysis (LMM) $p > 0.05$ between REACH versus usual care at any timepoint for all variables. β: beta regression coefficient, CI: Confidence Interval, MIP: maximum static inspiratory pressure, MEP: maximum static expiratory pressure, FEC: Functional exercise capacity, expressed in total steps per 2 minutes (two-minute step test), HGS: handgrip Strength, TMST: Two-Minute Step Test, Kg: kilogram
