# Supplementary material for: Course of recovery of respiratory muscle strength and its associations with exercise capacity and handgrip strength: A prospective cohort study among survivors of critical illness
Source: PLoS One. 2023 Apr 13;18(4):e0284097. doi: 10.1371/journal.pone.0284097 (PMC10101425; doi:10.1371/journal.pone.0284097)
Supplement: S1 Fig — MIP: Maximum inspiratory pressure, MEP: Maximum expiratory pressure. (PDF) [file pone.0284097.s004.pdf]

S1 Figure: Sensitivity analysis: course of recovery total sample versus complete cases (primary outcomes)

MIP % predicted, course over time: total N (n=59) versus complete cases (n=30)

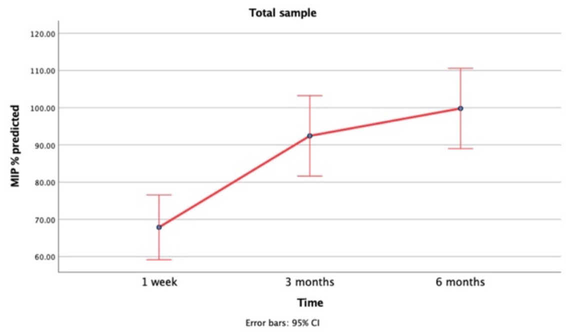

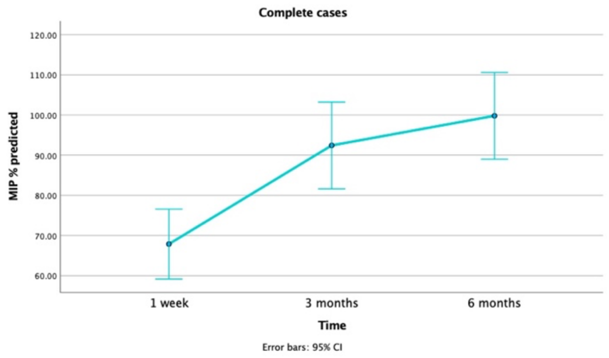

MEP % predicted, course over time: total N (n=59) versus complete cases (n=30)

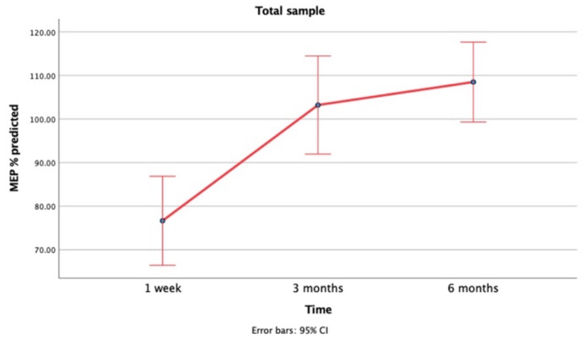

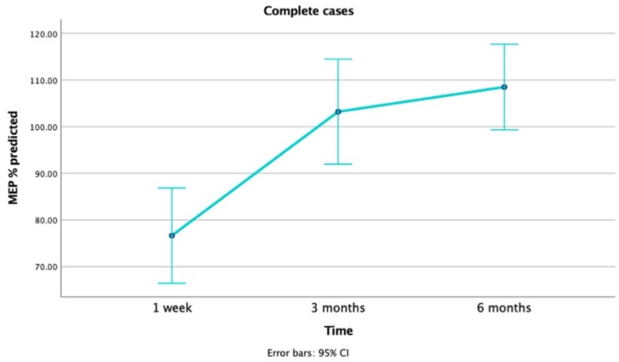

MIP: Maximum inspiratory pressure, MEP: Maximum expiratory pressure
