# Supplementary material for: Course of recovery of respiratory muscle strength and its associations with exercise capacity and handgrip strength: A prospective cohort study among survivors of critical illness
Source: PLoS One. 2023 Apr 13;18(4):e0284097. doi: 10.1371/journal.pone.0284097 (PMC10101425; doi:10.1371/journal.pone.0284097)
Supplement: S2 Fig — HGS: Handgrip strength, TMST: Two-minute step test. (PDF) [file pone.0284097.s005.pdf]

S2 Fig. Sensitivity analysis: course of recovery total sample versus complete cases (secondary outcomes)

HGS % predicted, course over time: total N (n=59) versus complete cases (n=30)	
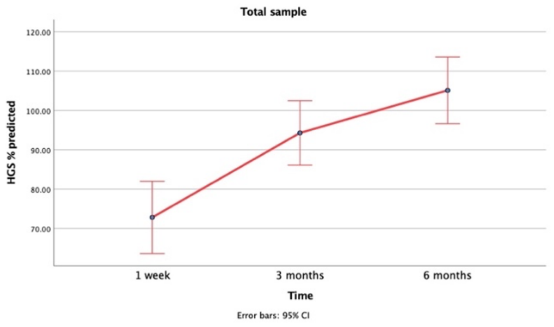	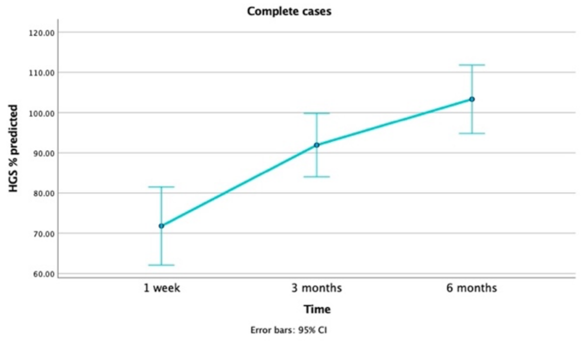
TMST step count, course over time: total N (n=59) versus complete cases (n=30)	
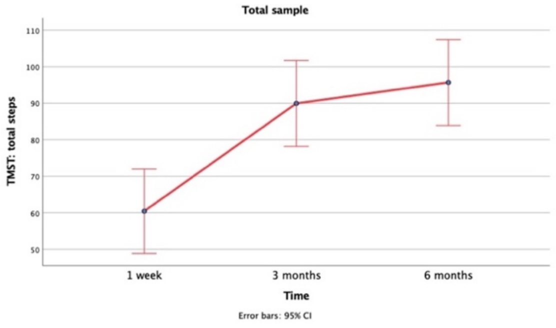	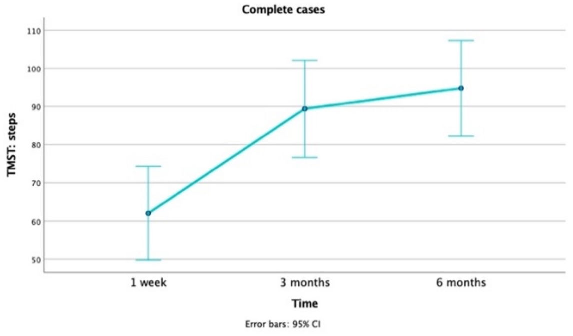

HGS: Handgrip strength, TMST: Two-minute step test
