# Supplementary material for: Course of recovery of respiratory muscle strength and its associations with exercise capacity and handgrip strength: A prospective cohort study among survivors of critical illness
Source: PLoS One. 2023 Apr 13;18(4):e0284097. doi: 10.1371/journal.pone.0284097 (PMC10101425; doi:10.1371/journal.pone.0284097)
Supplement: S3 Fig — MIP: Maximum inspiratory pressure, MEP: Maximum expiratory pressure, FEC: Functional exercise capacity, TMST: Two-minute step test, HGS: Handgrip strength, Kg: kilogram. T0: baseline, T1: 3 months follow up, T2: 6 months follow up. (PDF) [file pone.0284097.s006.pdf]

S3 Fig. Sensitivity analysis: Course of recovery REACH versus Usual Care (all outcomes)

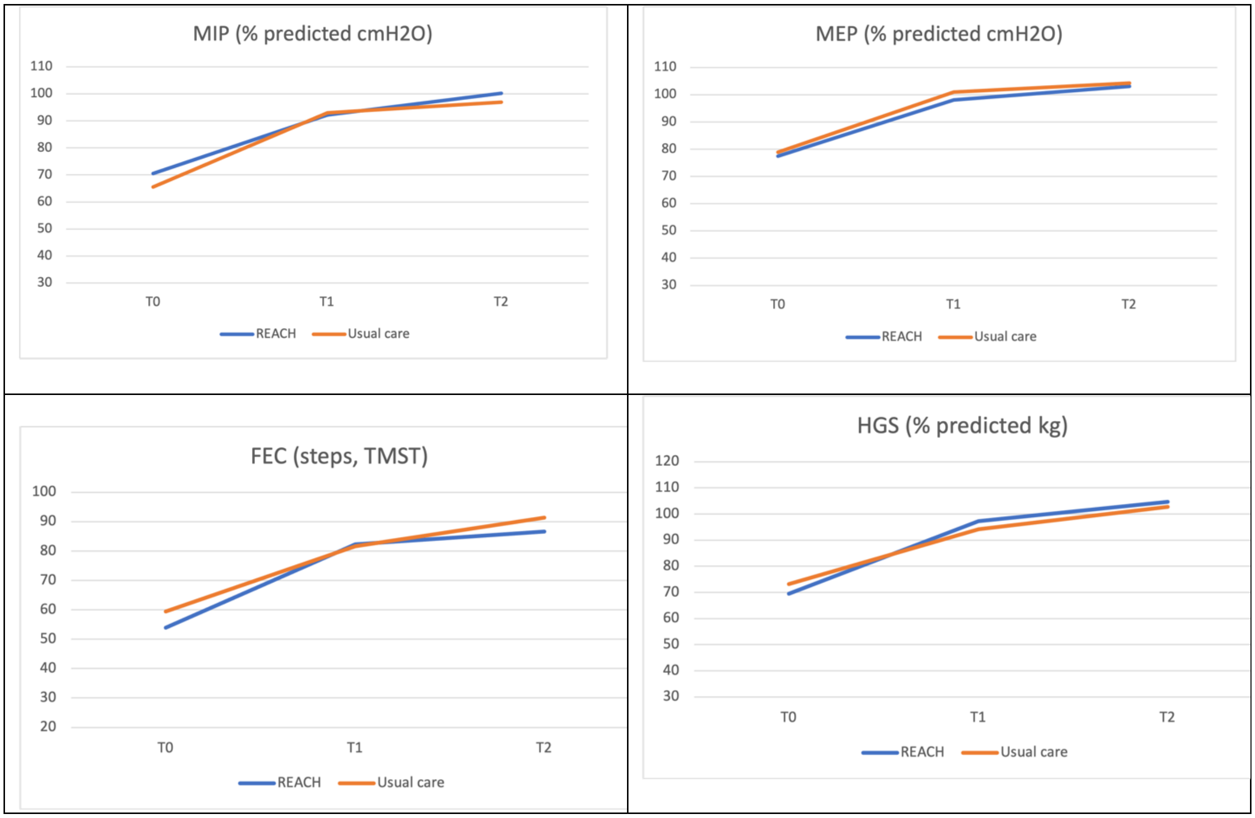

MIP: Maximum inspiratory pressure, MEP: Maximum expiratory pressure, FEC: Functional exercise capacity, TMST: Two-minute step test, HGS: Handgrip strength, Kg: kilogram. T0: baseline, T1: 3 months follow up, T2: 6 months follow up.
