# Supplementary material for: Course of recovery of respiratory muscle strength and its associations with exercise capacity and handgrip strength: A prospective cohort study among survivors of critical illness
Source: PLoS One. 2023 Apr 13;18(4):e0284097. doi: 10.1371/journal.pone.0284097 (PMC10101425; doi:10.1371/journal.pone.0284097)
Supplement: Attachment — Submitted filename: Response to reviewers.docx [file pone.0284097.s007.docx]

PONE-D-22-14900 Response to Reviewers

18-09-2022

Dear editor and reviewers,

First, we are grateful with the positive comments of the reviewers regarding the novelty, and the potential impact of our study and potential publication in PLOS ONE.

We thank the reviewers for their valuable comments and the editor to allow us to submit a revised version of our manuscript titled: *Course of recovery of respiratory muscle strength and its associations with exercise capacity and handgrip strength: a prospective cohort study among survivors of critical illness.*

With our revised manuscript we submit a point-by-point response to the academic editor and to the reviewers.

General comments from the editor:

1. We have addressed the style requirements as requested.
2. The DOI for the repository where the data to our study is held, is made available in the manuscript (10.21943/auas.20798332). Data sheets will be placed in this repository upon acceptance of the manuscript for publication.
3. No changes to the reference list were made.

Reviewer #1:

1. Related to the singular importance of the sex variable as applied in the longitudinal analysis in which we investigated crude and adjusted associations between PImax/PEmax and handgrip strength and exercise capacity. The reviewer addresses an important concern with the modification of the effect in the associations when adjusting for sex. The way these results were presented by us, does not provide clarity on this modification. We have re-run our Mixed Model Analysis through stratification by gender (as suggested by reviewer 1) and are presenting the crude and adjusted associations for three categories: the entire study population, the male group, and the female group. Indeed, some differences in the (strength of the) associations become evident. We have adjusted the statistical analysis methods, some of the written results and table 4 accordingly and have briefly reflected upon the implications in the discussion.
2. Standardizing the presentation of the confidence intervals using ‘to’ in the entire manuscript, as suggested, has been applied throughout.

Reviewer #2:

1. This reviewer correctly highlights the importance of differentiating between patients who have received mechanical (invasive) ventilation and patients who have received non-invasive ventilation, as their outcome is likely to be different. We would like to clarify that all our patients received invasive mechanical ventilation, in fact patients were only eligible to participate had they received invasive mechanical ventilation. We have clarified this in the manuscript text.
2. Clarification of the measurement methods of the TMST, including how the ‘set criterion height’ was established and which contraindications were applied: these are again very valuable suggestions - we have clarified in the manuscript text and in the legend of Fig 1.
3. Page 11, line 261: replace PImax with PEmax - resolved
4. Page 12, line 281, add S3 Fig to Supporting information: S1-S3 Table, S1 Fig, S2 Fig. - resolved
5. Check and resolve the inconsistency between the MIP at T0 in the abstract and in Table 2. - resolved

We thank the editor again for allowing us to revise and resubmit our manuscript based on the reviewer comments.

Sincerely,

Dr. Mel E. Major,

Dr. Maarten A. van Egmond, Dr. Daniela Dettling-Ihnenfeldt, Dr. Stephan P.J. Ramaekers, Prof. Dr. Raoul H.H. Engelbert and Dr. Marike van der Schaaf.
